# A robust protocol for efficient generation, and genomic characterization of insertional mutants of *Chlamydomonas reinhardtii*

**DOI:** 10.1186/s13007-017-0170-x

**Published:** 2017-04-03

**Authors:** Steve V. Pollock, Bratati Mukherjee, Joanna Bajsa-Hirschel, Marylou C. Machingura, Ananya Mukherjee, Arthur R. Grossman, James V. Moroney

**Affiliations:** 1 Department of Biological Sciences, Louisiana State University, Baton Rouge, LA 70803 USA; 2 Department of Plant Biology, Carnegie Institution for Science, Stanford, CA 94305 USA

## Abstract

**Background:**

Random insertional mutagenesis of *Chlamydomonas reinhardtii* using drug resistance cassettes has contributed to the generation of tens of thousands of transformants in dozens of labs around the world. In many instances these insertional mutants have helped elucidate the genetic basis of various physiological processes in this model organism. Unfortunately, the insertion sites of many interesting mutants are never defined due to experimental difficulties in establishing the location of the inserted cassette in the Chlamydomonas genome. It is fairly common that several months, or even years of work are conducted with no result. Here we describe a robust method to identify the location of the inserted DNA cassette in the Chlamydomonas genome.

**Results:**

Insertional mutants were generated using a DNA cassette that confers paromomycin resistance. This protocol identified the cassette insertion site for greater than 80% of the transformants. In the majority of cases the insertion event was found to be simple, without large deletions of flanking genomic DNA. Multiple insertions were observed in less than 10% of recovered transformants.

**Conclusion:**

The method is quick, relatively inexpensive and does not require any special equipment beyond an electroporator. The protocol was tailored to ensure that the sequence of the Chlamydomonas genomic DNA flanking the random insertion is consistently obtained in a high proportion of transformants. A detailed protocol is presented to aid in the experimental design and implementation of mutant screens in Chlamydomonas.

**Electronic supplementary material:**

The online version of this article (doi:10.1186/s13007-017-0170-x) contains supplementary material, which is available to authorized users.

## Background

Over the past decade, *Chlamydomonas reinhardtii* (hereafter referred to as Chlamydomonas) has been successfully used as a model system to help answer biological questions related to a wide variety of cellular processes. With a sequenced genome and a growing experimental toolbox to facilitate large-scale forward and reverse genetic studies, this unicellular microalga now provides an even stronger functional genomics template for the further dissection of biological processes, and both metabolic and regulatory pathways. Experimental work using Chlamydomonas will not only contribute to our increased understanding of its own physiology and biochemistry, but will continue to reveal the genetic basis of similar processes in other organisms such as bacteria, fungi, vascular plants, animals and even humans.

A valuable resource for studying biological processes is the availability of stable mutations that disrupt key genes that encode components of those processes. In Chlamydomonas, insertional mutagenesis has been routinely used for this purpose, and with great success. A large number of transformants can be generated using this technique with the goal of tagging a single functional gene within the nucleus of each transformant. When transforming nuclear DNA, a short DNA cassette consisting of an antibiotic resistance gene marker flanked by endogenous promoter and terminator sequences is used to ensure optimum marker gene expression and prevent transcriptional read through. In Chlamydomonas, the absence of homologous recombination in the nuclear genome means that insertion of the antibiotic cassette occurs at random genomic sites. Identification of the location of the insert in the genome is therefore pivotal to the success of this method. In forward genetic studies, the position of the inserted DNA in mutants with phenotypes of interest would help identify genes disrupted in the transformants. However, this approach could also be valuable for reverse genetic screens since there are many Chlamydomonas genes, both with known and unknown functions, in which knockouts might not result in readily discernible phenotypes.

With the current availability of a sequenced and largely annotated nuclear genome [1, 2], a relatively short sequence flanking the genomic insert is often provides enough information to use the genomic database to identify the disrupted genomic locus. Several techniques were successfully used in the past to identify insertion sites within the genome. These involved modified protocols for plasmid rescue [3], Thermal asymmetric interlaced PCR or TAIL PCR [4], Restriction enzyme site-directed amplification PCR or RESDA-PCR [5], 3′-Rapid Amplification of cDNA ends or 3′RACE [6] and Site Finding PCR [7]. Recently, a high throughput Mme1-based Insertion site Sequencing strategy for Chlamydomonas insertional mutants, called ChlaMmeSeq [8, 9], was used for the simultaneous screening of large numbers of mutagenic insertion sites. Keeping in mind the available technology used with varying degrees of success in Chlamydomonas, this study proposes the use of a different protocol aimed at the successful recovery of genomic regions flanking an insert within transformants generated in large scale insertional mutagenesis efforts. This protocol is based on an adaptor-linked PCR method that has been modified and refined for success with Chlamydomonas. It is robust, time efficient, and identifies the location of inserts in the majority of transformants. This method also detects the number of insertions and their direction, and any deletions/rearrangements at the insertion site.

Adaptor linked PCR has been used with prokaryotes [10] and eukaryotes for genome walking, as well as to identify the location of T-DNA inserts in the Arabidopsis genome [11]. This procedure usually involves restriction and blunting of genomic DNA, followed by ligation of an asymmetric adaptor DNA oligonucleotide that has been modified to prevent self-ligation. The adaptor linked to the ends of genomic DNA provides a template for designing primers based on known sequences of the adaptor and of the inserted cassette for a series of nested PCRs with varying stringency. These PCRs produce DNA fragments that are sequenced and aligned to the nuclear genome sequence. In this study, the adaptor linked PCR method was modified for efficient use in determining insert locations in Chlamydomonas transformants derived from a large-scale mutagenesis effort. Several combinations of restriction enzymes and PCR conditions were tested to provide a robust and relatively fail-safe method of determining insert location on the Chlamydomonas nuclear genome.

A detailed protocol is provided to aid researchers interested in high throughput determination of insert locations with a population of transformants. Several recommendations in areas ranging from primer design to the use of combinations of restriction enzymes that might further increase the probability of insert recovery are presented. Many shortcuts have also been suggested such as one step digestions and ligations, rapid methods for agarose gel electrophoresis, and the use of shorter PCR cycles to reduce both time and cost. The execution of the technique proposed in this study will contribute to both the generation of genome wide mutant libraries and the characterization of the insertion sites.

## Methods

### Generation of the insertional DNA fragment

**Fig. 1:**
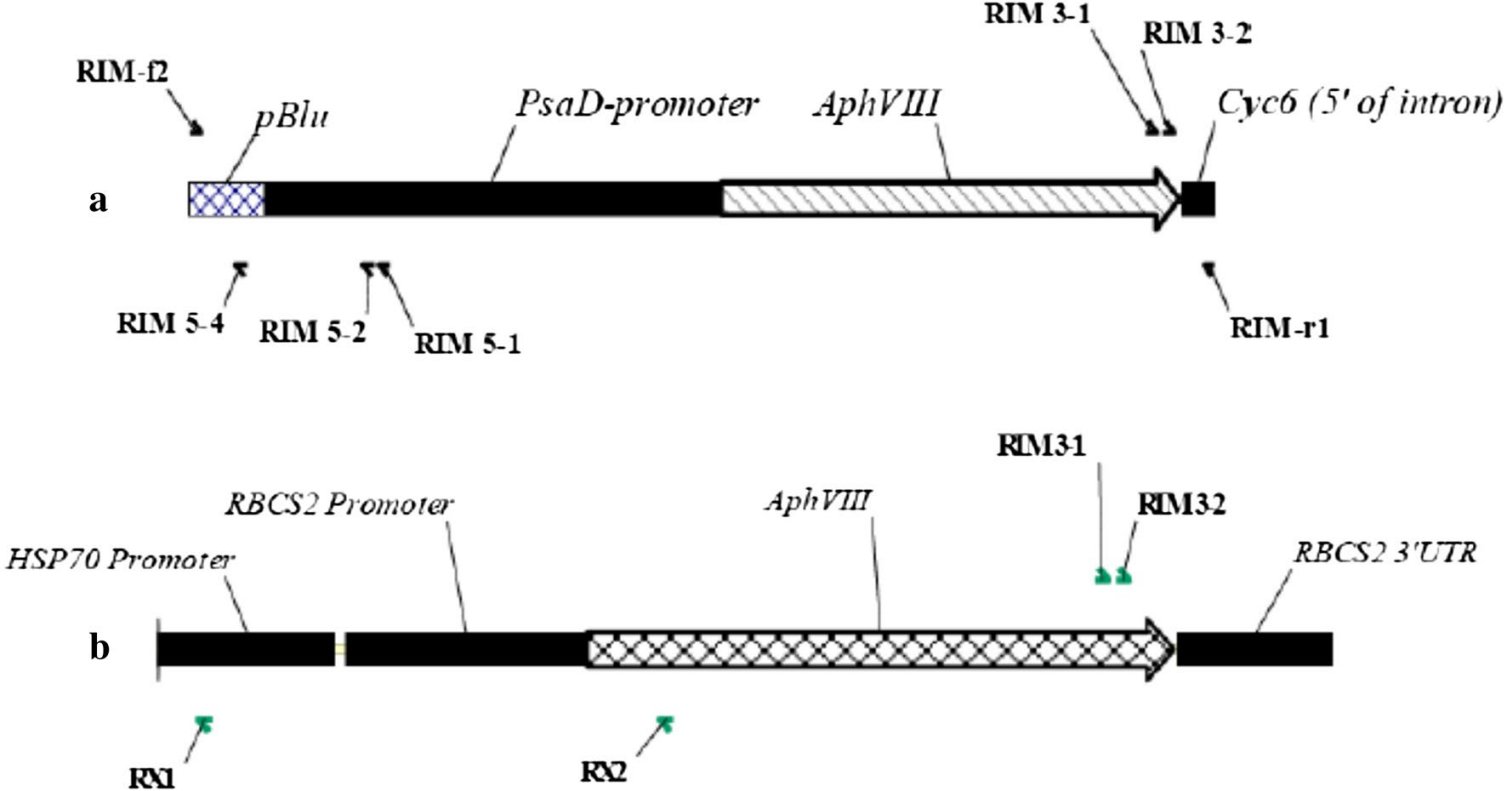
The paromomycin cassette. Two different methods were used to generate the paromomycin cassette used in these experiments. **a** PCR generated cassette. The paromomycin resistance cassette used to generate paromomycin resistant strains, and the primers used to amplify the cassette (RIM-f2, and RIM-r1) from pSL72. The other primers indicated in the figure were used to amplify genomic DNA flanking the site of the insertion. **b** Restriction digested generated cassette. The paromomycin cassette from the pSL18 plasmid

**Fig. 2:**
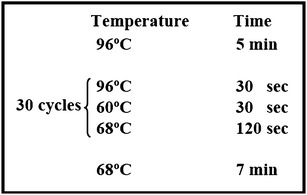
The temperature cycling protocol used to amplify the insertional DNA fragment

Two methods were used to generate the cassette used for transformation. One used digestion from the pSL18 plasmid and the other used PCR amplification. The PCR generated DNA fragment was amplified from vector pSL72 [12] and transformed into *C. reinhardtii* strain D66 (*nit2*; *cw15; mt* ^+^) to confer resistance to paromomycin. Primers, RIM-f2 and RIM-r1 (see Table 1 for primer sequences), were used to amplify a DNA fragment with 122 bp of the bacterial pBluescript vector, 803 bp of the Chlamydomonas *PSAD* promoter, 811 bp of the *AphVIII* coding sequence from *Streptomyces rimosus* [13] and 58 bp of the 5′ end of the second intron of the *CYC6* gene of Chlamydmonas (Fig. 1a). The Expand Long Template PCR System (Roche; CAT. #11681834001), a mixture of thermostable TAQ DNA polymerase and Tgo (a proofreading DNA polymerase) and approximately 10 ng of the pSL72 plasmid template were used to amplify the 1812 bp DNA fragment. The composition of a single PCR reaction is shown in Table 2. The temperature cycling protocol is shown in Fig. 2. Alternatively, the cassette was obtained by restriction digestion of the pSL18 plasmid. *Xho*I and *Nhe*I (NEB) were used to digest the plasmid. The resulting fragment contained the HSP70-RbcS2 dual promoter followed by the paromomycin gene and the *RBCS2* terminator (Fig. 1b).

**Table 1:** The primers used for this work

Primer	Description	Sequence 5′–3′
RIM-f2	Used to amplify cassette	TGT GTG GAA TTG TGA GCG G
RIM-r1	Used to amplify cassette	CTT TCC ATC GGC CCA GCA
RIM 3-1	3′ insert primer	CGG TAT CGG AGG AAA AGC TG
RIM 3-2	3′ insert primer	GCT GTT GGA CGA GTT CTT CTG
RIM 5-1	5′ insert primer	TTC CAA GCG ATC ACC AGC AC
RIM5-2	5′ insert primer	GCT GGC ACG AGT ACG GGT TG
RIM5-4	5′ insert primer	AGC TTT TGT TCC CTT TAG TG
AP1	Adaptor primer	GTA ATA CGA CTC ACT ATA GAG T
AP2	Adaptor primer	ACT ATA GAG TAC GCG TGG T
RX1	5′ insert primer pSL18	GCC CTC ATA GCC CGC CAA ATC AG
RX2	5′ insert primer pSL18	AAG CCG ATA AAC ACC AGC CC

**Table 2:** Composition of the 50 µL PCR reaction mixture used to amplify the 1812 bp insertional DNA fragment

Component	Volume
dH_2_O	37.5 µL
10× polymerase buffer #3	5 µL
2.5 mM dNTP mix	4 µL
RIM-f2 (20 µM)	1 µL
RIM-r1 (20 µM)	1 µL
pSL72 plasmid template	1 µL^a^
Polymerase mix (5 U µL^−1^)	0.5 µL

^a^Containing approx. 10 ng plasmid DNA

### Preparation of the DNA insert

After electrophoresis in an 0.8% (W/V) agarose gel, the DNA fragments were purified from the gel by excising the 1812 bp fragment (PCR generated) or the 1813 bp fragment (restriction generated) with a razor blade (visualized with ethidium bromide and a low energy UV light). DNA from the gel slices were purified using a gel extraction kit (Qiagen). The purified DNA was filter sterilized and quantified by comparing the fluorescence of the DNA to that of a known DNA mass ladder (*Hind*III digested lamda DNA (New England Biolabs); or a 1 Kb ladder (New England Biolabs).

### Preparing chlamydomonas cells for transformation

500 mL of TAP medium [14] was inoculated with a 4 mm spherical scraping (a small green pea volume equivalent) of D66 cells from a TAP agar plate. When cells reached a density of approximately 3 × 10^6^ cells mL^−1^ they were harvested by centrifugation at 2500×*g* and resuspended in a final volume of 3.5–4.0 mL TAP containing 60 mM sorbitol to achieve a final cell concentration of 2–4 × 10^8^ cells per mL. Resuspended cells were transferred to a 15 mL Corning centrifuge tube (Corning #430790) and placed on ice. Electroporation cuvettes (Bio-Rad #165-2091) with a gap-width of 0.4 cm were used for electroporation. 250 µL of cells was transferred to a sterile cuvette with no added DNA, labeled “No DNA control”, and placed on ice. Approximately 2.5 µg of the prepared insert DNA fragment was added to the remaining 3.25–3.75 mL of suspended cells, gently inverted 5–6 times to make the DNA/cell mixture homogeneous and then placed on ice. 250 µL aliquots of the DNA/cell suspension were added to the sterile electroporation cuvettes, yielding approximately 14 transformation reactions. All cuvettes were placed on ice for 10–20 min before electroporation. This step is critical in obtaining a pulse rate that does not lyse the cells (see below). Care must be taken to ensure the cells do not warm up before the pulse is generated.

### Electroporation of chlamydomonas

As stated above, 250 µL containing between 0.5 × 10^8^ and 1 × 10^8^ cells with 180 ng of the paromomycin cassette were electroporated using the Bio-Rad Gene Pulser II system, as modified from the method reported by Shimogawara et al. [15]. One significant modification was that no carrier DNA was added. A voltage setting of 0.8 kV, a capacitor setting of 25 µF, and no shunt resistor, was used for electroporation. The measured pulse time, an excellent predictor of the success of the electroporation transformation, generally ranged from 10 to 13 ms. If the pulse time was shorter, the transformation efficiency decreased approximately 100-fold as cells were lysed during the pulse. The cuvettes were placed at room temperature for 5 min following electroporation and prior to transfer to overnight recovery medium.

### Overnight recovery of electroporated cells

Within 30 min of electroporation, the mixture from each cuvette was transferred using a 200 µL wide orifice pipet tip (E&K #3502-R96S) (to prevent shearing of the cells) to 10 mL of TAP medium containing 60 mM sorbitol in a 15 mL Corning centrifuge tube (Corning #430790). The tubes were placed in low light (10–20 µmol photons m^−2^ s^−1^), gently rocked to keep the cells suspended, and allowed to recover overnight (12–16 h). Used electroporation cuvettes were rinsed with water and stored in 100% ethanol until required for another round of transformations. As long as the cuvettes did not crack they were reused several times. Cuvettes were dried in a sterile hood immediately before use.

### Plating recovered electroporated cells on selective medium

**Fig. 3:**
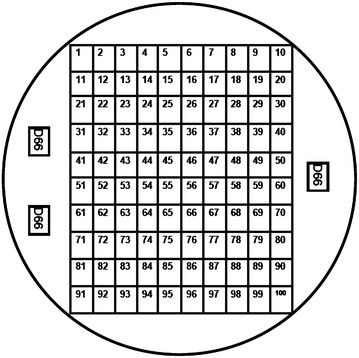
The 10 × 10 grid template used to plate the paromomycin transformants. The D66 squares are the untransformed parental control blocks

Petri dishes with solid TAP medium containing 1.5% agar (W/V) and 7.5 µg mL^−1^ of paromomycin sulfate (Sigma # P5057; stock of 100 mg mL^−1^ dissolved in dH_2_O, filter sterilized, and frozen) were prepared one day in advance of plating. The 10 mL of recovered cells were harvested by centrifugation in an IEC swing-out clinical centrifuge at maximum speed for 1 min and resuspended in 80–100 µL of TAP medium. The entire mixture was gently spread, using a bent glass rod, on a single TAP plus paromomycin plate and allowed to dry in a transfer hood. Dried plates were placed in moderate light (50–80 µmol photons m^−2^ s^−1^). Within 2 days of plating the majority of the cells began to die and after 4 days small colonies were noted under a dissecting microscope. After 1 week the colonies were large enough to pick with sterile pointed toothpicks onto a screening plate using a 10 × 10 grid (Fig. 3). The typical yield of paromomycin resistant transformants varied from 150 to 300 colonies per plate. This corresponds to roughly 4 transformants per 10^6^ cells. Once accustomed to picking colonies, a single researcher can “easily” pick 500 colonies in 2 h. A self-closing pair of tweezers made holding toothpicks more comfortable and allowed the user to use both ends of the toothpicks. Used toothpicks were reused after autoclaving and drying.

### Genomic DNA preparation

Total DNA was isolated from 50 mL of mutant cells grown in TAP medium under continuous low light (50–80 µmol photons m^−2^ s^−1^) according to Newman et al. [16] with several modifications. Briefly, cells were pelleted by centrifugation at 2500×*g* in 50 mL sterile centrifuge tubes (Corning #430828) and resuspended in 400 µL of dH_2_0 in two 1.5 mL Eppendorf tubes and 800 µL disruption buffer containing SDS was added (2% SDS, 400 mM NaCl, 40 mM EDTA, 100 mM Tris–HCl, pH 8.0). The nucleic acids were extracted three times using a phenol/chloroform/isoamyl alcohol mixture until the interface of the inorganic and organic layers contained no residual protein residue. The aqueous phase was then extracted a final time with chloroform. Nucleic acids were then precipitated with two volumes of ethanol and washed twice with 70% ethanol. The pellet was air-dried for 10 min and dissolved in 100 µL of TE (10 mM TRIS, pH7.5, 1 mM EDTA). See Additional file 1 for a more detailed description of the DNA preparation protocol.

### Preparation of blunt-ended restriction digest fragments of genomic DNA

200 ng of genomic DNA was digested with a mixture of *Ale*I (10 units), *Nae*I (10 units), *Pml*I (10 units) and *Pvu*II (1 unit) restriction endonucleases (New England Biolabs) in a volume of 100 µL in NEB buffer 2 supplemented with 100 µg mL^−1^ BSA. The reactions were incubated at 37 °C for 16–18 h. These four restriction endonucleases were chosen because they do not recognize sequences in the insertion DNA fragment and because they recognize sequences that occur, on average, every 200 bps in the *C. reinhardtii* genome, creating blunt-ended fragments. The last hour of the reaction was supplemented with 1 µL RNAse to degrade RNA. After the incubation, 5 µL of digested genomic DNA was separated by agarose gel electrophoresis to verify that the digestions were complete, which was observable as a smear of DNA from approximately 1 to 6 kb on a 1% agarose gel. Digested DNA was extracted once with an equal volume (95 µL) of phenol:chloroform:isoamyl alcohol (25:24:1 v/v), and once with chloroform:isoamylalcohol (24:1 v/v), precipitated with three volumes (285 mL) of ethanol, the pellet washed once with ice-cold 80% (V/V) ethanol, air-dried for 10 min and then resuspended in 20 µL of TE.

### Adaptor preparation

**Fig. 4:**
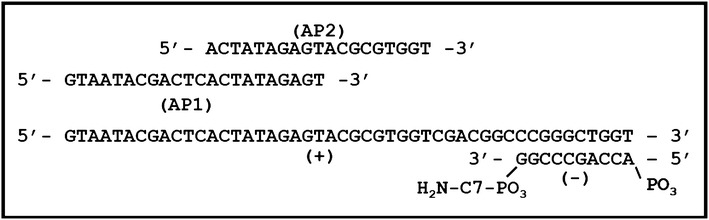
The adaptor used to obtain the flanking DNA. The blunt-ended asymmetric adaptor, consisting of a 48 bp positive strand (+) and a 10 bp negative strand (−), and the two adaptor primers (AP1 and AP2) aligned to depict where they will bind after the negative strand of the adaptor is extended in the first round of the primary PCR reaction

A blunt-ended adaptor consisting of a 48 bp DNA oligo, designated plus strand, and a 10 bp oligonucleotide, designated negative strand, were procured from Integrated DNA Technologies. The plus strand, 5′-GTA ATA CGA CTC ACT ATA GAG TAC GCG TGG TCG ACG GCC CGG GCT GGT-3′, was procured (250 nmol level) and HPLC purified by the manufacturer. The minus strand, 5′-ACC AGC CCG G-3′, was procured at the (100 nmol level), with a 3′ C3 spacer to prevent polymerase extension, and 5′ phosphorylation to permit ligation, and HPLC purified. The two strands were each dissolved in STE (10 mM Tris (pH 8.0), 50 mM NaCl, 1 mM EDTA) at a concentration of 50 µM and 25 µL of each strand was mixed together and placed in a thermocycler at 95 °C. The thermocycler was programmed to gradually cool to 4 °C over a period of approximately 3 h to allow the two strands to anneal to form a double stranded asymmetric blunt-ended adaptor (Fig. 4). The resulting adaptor is stable and can be stored at 4 °C or frozen. To prevent denaturation of the adaptor, it was kept cool during handling.

### Ligation of the adaptor to the digested DNA

The adaptor was ligated to the digested genomic DNA overnight (16–20 h) at 16 °C. The ligation reaction consisted of 2.0 µL of adaptor, 4 µL of digested genomic DNA, 2 µL of 10× New England Biolabs ligase buffer, 1 µL of T4 DNA ligase (NEB #M0202S). The reaction was stopped by incubating the mixture at 80 °C for 20 min. Seventy µL of TE was added to the reaction before use as template during the PCR amplification.

### Alternative one-step restriction digest and ligation reaction

Alternatively, the genomic DNA was restricted and ligated to the adaptor in a one-step reaction. The one step reaction was performed in the restriction enzyme buffer (NEB#2) with the addition of 10 µM ATP. The reaction was performed overnight at room temperature.

### PCR amplification from the insertion to the flanking adaptor

**Fig. 5:**
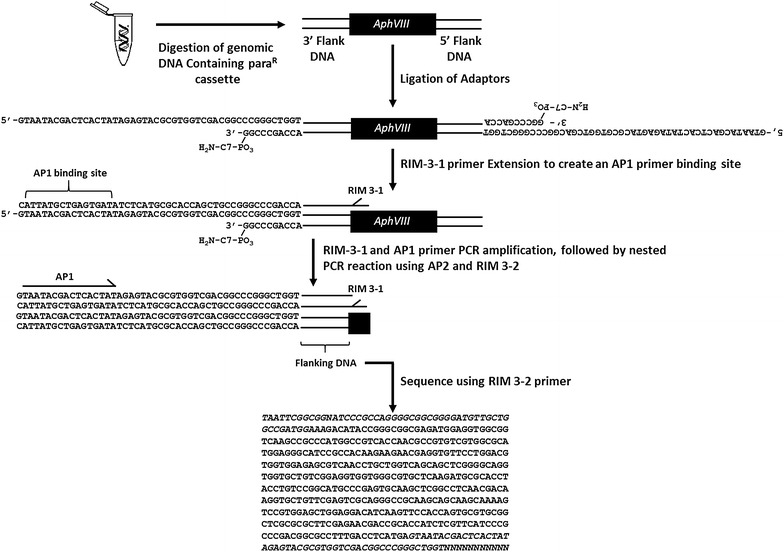
The adaptor PCR method. A flow-chart depicting the adaptor-mediated PCR method to obtain DNA sequence flanking the insertion of the paromomycin resistance cassette (*AphVIII*). The *bold* text in the final sequence highlights the 3′ end of the insertion DNA, and the *bold/italicized* text depicts the 48 bp sequence that is sometimes present in shorter DNA fragments

**Fig. 6:**
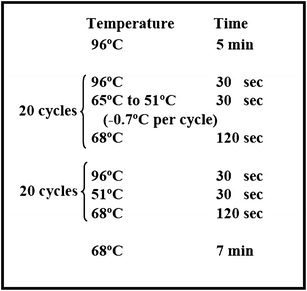
The touch-down PCR protocol. The touch-down temperature cycling protocol used to amplify DNA flanking the insertional DNA fragment

Primary and nested PCR reactions were used to amplify the genomic DNA flanking the insertion of the paromomycin resistance cassette. By using primer sets directed out from the 5′ or 3′ ends of the insert, it was possible to amplify DNA flanking both sides of the insert (Fig. 5). The primary reaction utilized an insert specific primer (RIM3-1; or RIM5-1) and an adaptor primer (AP1) (see Table 1 for lists of primers). RIM3-1 and RIM5-1 were used to amplify the 3′ and 5′ flanking DNA respectively (Fig. 1). To enrich for amplification from the paromomycin insertion the AP1 primer binding site was only generated after the RIM3-1 primer extended the 10 bp strand of the adaptor to yield an adaptor sequence for AP1 primer binding. A touch-down PCR protocol using Expand Long Template PCR System (Roche; CAT. #11681834001) was utilized for the primary and nested PCR reactions (Fig. 6). The primary reaction was diluted 50-fold by resuspending 1 µL of the primary reaction in 49 µL of dH_2_O and vortexed to mix. The nested PCR reaction was then performed using the nested primers RIM3-2 (or RIM 5-2) and the adaptor primer AP2 using the same cycling parameters used in the primary reaction. In some instances the use of an additional nested PCR reaction using RIM 5-4 was necessary to obtain a 5′ flanking DNA fragment. The RIM5-4 primer was designed to bind to the portion of the cassette arising from pBluescript. All DNA fragments amplified by the above procedure were sequenced using the last insertion specific primer (RIM 3-2, or RIM5-4).

## Results and discussion

### Adaptor PCR

**Fig. 7:**
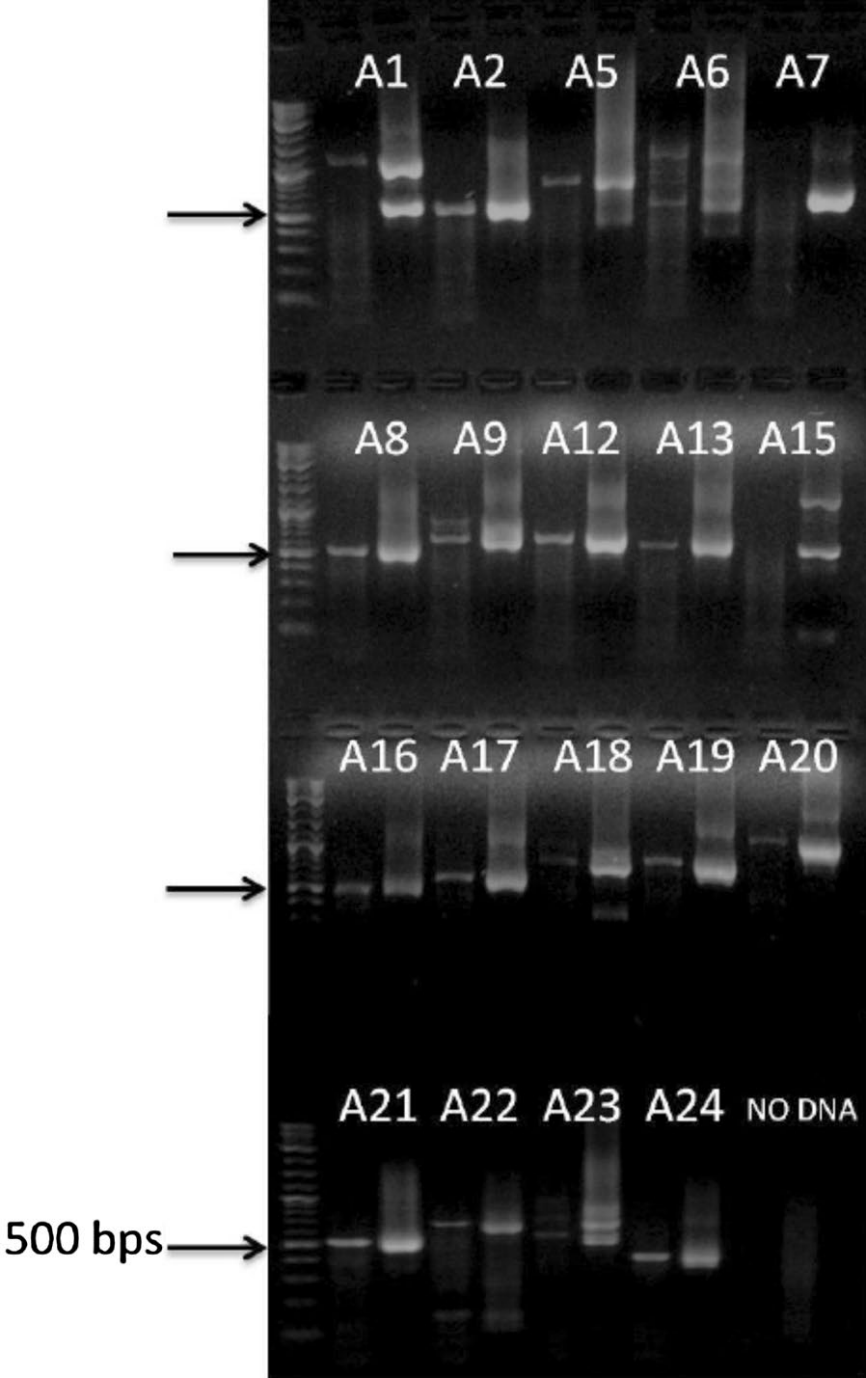
Representative results using this adaptor PCR method. Representative results from several insertional mutants (A1–A24) following the described protocol. Agarose gel electrophoresis was used to visualize the products of the primary and secondary PCR reactions respectively for each mutant. A diagnostic step-down in fragment size was indicative of a positive result as the nested primers amplified the target DNA from the mutants

In this investigation, 30,000 insertional mutants were selected following transformation with the paromomycin resistance conferring cassette. After this selection, colonies were screened for growth on high and low CO_2_ and 211 colonies showing a growth deficiency only under low CO_2_ conditions were chosen for further analysis. Adaptor PCR was performed on the genomic DNA of the mutants. The majority of the mutants yielded a single PCR product, but some also produced two or more products. In most cases, the length of the flanking DNA ranged from 150 bp to 1200 bp while some fragments >2000 bps were obtained. A representative agarose gel is shown in Fig. 7. Fragments were excised from the gels and sequenced. The majority of the sequenced fragments contained the 3′ end of the paromomycin resistance cassette followed by Chlamydomonas genomic DNA. Some PCR products were too short to map accurately to the Chlamydomonas genome (for instance, fragments less than 15 nucleotides in length), and some contained only the paromomycin vector sequence. However, we were able to map over 74% of the sequences (156 out of 211 independent inserts) to the Chlamydomonas genome. From the 156 colonies where the DNA flanking the insert was identified, 36 colonies were chosen for further molecular analysis. This analysis included mapping both ends of the insert as well as checking for DNA deletions in the regions flanking the paromomycin cassette. When both ends of the insert were mapped we found that 75% of the mapped inserts (27/36) represented simple insertions of the resistance cassette resulting in no genomic deletion of > 10 bp. Of the remaining 25% of the colonies (9/36), we were unable to map the 3′ end of the insert to the same genomic region identified at the 5′ end. Thus, in some instances a Chlamydomomas genomic fragment is inserted between the cassette and the true genomic location. This result is similar to that of Zhang et al. [8] who mapped inserts using the ChlaMmeSeq method. Overall, the results show that 75% of the insertional mutants generated by our mutagenesis resulted in a simple insertion that sometimes had a small DNA deletion.

This report details a method that enables researchers to generate insertional mutants and to successfully identify the location of the paromomycin resistance cassette in the Chlamydomonas genome over 75% of the time. The method is rapid, fairly inexpensive and does not require exotic equipment, except for the electroporator. This method differs significantly from other methods which primarily use TAIL PCR or a modification of that method [4, 5]. First, the transformation method described in this report was electroporation versus glass beads, to reduce genomic deletions. Secondly, a DNA cassette was used containing only the paromomycin resistance gene instead of a plasmid [4, 5]. The use of a cassette makes it easier to identify the ends of the inserted DNA and makes the adaptor method a viable choice for researchers. In addition, a combination of restriction enzymes is described that enhances the likelihood of cutting the flanking genomic DNA within 1000 bp of the insert, increasing the success of obtaining a PCR fragment long enough to identify the site of insertion. Finally, adaptor PCR was used instead of TAIL PCR, which offers an additional tool to use if a researcher is having trouble with one method. Recently, a library of Chlamydomonas insertional mutants became available to researchers [9]. This major advance will allow researchers to order Chlamydomonas strains containing an insert in specific genes. However, for gene discovery, scientists will still need to generate and screen new insertional mutants in their own laboratories. After conducting these screens and selecting strains with the desired phenotype, researchers need to be able to identify the disrupted gene. Furthermore, the new insertion library is not complete (less than 40% of the genes covered by more than one allele, and only a small number of the mutants have been validated). Finally, the method for generating insertional mutants described here will result in a high percentage of the colonies having single, simple insertions with few large genomic deletion. This transformation procedure also yields a relatively low number of strains with multiple DNA insertions. We found that multiple inserts were present in 11 out of the 156 mutants (~6%), although some insertions will not be detected using this method. Zhang et al. [8] observed that about 15% of the paromomycin resistant transformants had more than one insert when using a similar transformation protocol. We also observed very few deletions of genomic DNA flanking the inserts. One disadvantage of the glass bead transformation method is that it sometimes results in large DNA deletions resulting in the loss of more than one gene, making the results much harder to analyze [17, 18]. The electroporation method appears to be less likely to cause these large deletions although deletions have been reported using this method also [19, 20]. While we did not find large deletions in the transformants that we characterized, we were unable to recover the other side of the insert in 25% of the colonies, which could indicate the occurrence of large deletions or rearrangements.

### Cosegregation of the DNA insert with the phenotype being studied

For any study using insertional mutagenesis, cosegregation of the insert with the desired phenotype must be genetically demonstrated. Genetic linkage was investigated in 15 strains generated using this method and paromomycin resistance cosegregated with the SLC phenotype about 40% of the time (6 of 15 transformants). Cells with an SLC phenotype grow normally on elevated CO_2_ concentrations but more slowly than wild-type cells at low CO_2_ concentrations. This rate of cosegregation is similar rates reported by others [4]. Since random insertion involves double strand DNA breaks the generation of insertions and point mutations is likely. Clearly it remains essential that a genetic analysis of any interesting insertional mutant be done before proceeding with a complete physiological characterization of the mutant. Tetrad analysis was used in this study but random spore analysis could also be used. It is also critical to demonstrate rescue of the mutant phenotype by introduction of a wild type copy of the disrupted locus.

### Earlier successes

Earlier versions of the transformation procedure described here were used to generate and characterize insertional mutants in two large scale experiments. In one study, over 30,000 insertional mutants were screened for aberrant responses to sulfur limitation (SAC) [21]. In a separate investigation, again over 30,000 insertional mutants were generated and screened for a ‘sick in low carbon dioxide’ phenotype (SLC). In some cases, mutants showing the desired phenotype were subjected to adaptor PCR to determine the genomic location of the paromomycin resistance cassette in their genomes. In the study in which the cells were screened for aberrant responses to sulfur limitation, a number of novels genes were discovered, including proteins involved in the responses of Chlamydomonas to sulfur deprivation [19, 21]. In the screen for mutants unable to grow photoautotrophically on low CO_2_, insertions in *CIA6* [22], bestrophin, *MITC11* and *LCI9* [23] and *CIA8* (Machingura, Bajsa-Hirschel and Moroney, unpublished) were identified using the forward genetics approach described by González-Ballester et al. [5]. No large deletions were observed in these studies.

## Conclusion

In this communication we have detailed a method to generate insertional mutants that will have mostly single simple DNA inserts. We have described an adaptor-PCR based method that reliably can identify the location of the cassette in Chlamydomonas. Using this method, we were able to identify the genomic DNA flanking the insertion over 75% of the time. The combination of employing the electroporation method to generate insertional mutants in conjunction with the adaptor method should provide researchers using Chlamydomonas an excellent chance to quickly generate and characterize useful insertional mutant strains at a relatively low cost.

## Additional file

**Additional file 1.** A simple Protocol for obtaining Chlamydomonas genomic DNA.
