# Supplementary material for: A robust protocol for efficient generation, and genomic characterization of insertional mutants of Chlamydomonas reinhardtii
Source: Plant Methods. 2017 Apr 3;13:22. doi: 10.1186/s13007-017-0170-x (PMC5376698; doi:10.1186/s13007-017-0170-x)
Supplement: Supplementary file 1 — Additional file 1. A simple Protocol for obtaining Chlamydomonas genomic DNA. [file 13007_2017_170_MOESM1_ESM.docx]

**Additional File 1.** A simple Protocol for obtaining Chlamydomonas genomic DNA.

Scrape cells off plate into 1.5 mL Eppendorf tube that contains 0.5 mL TEN buffer.

**OR**

Harvest 50 mL of cells grown in TAP and resuspend in approximately 400 μL dH_2_O and split into two 1.5 mL tubes.

- Resuspend vigorously by vortexing, spin for 10 sec. and aspirate off supernatant.
- Resuspend cells in 150 μL H_2_O on ice and add 300 μL of SDS-EB buffer, vortex to mix.
- Extract one to three times with 350 μL phenol:Chloroform:isoamyl alcohol (1:1) for few min by inverting, separate phases by centrifugation for 5 min., transfer aq. phase to a new tube.
- Extract once with 300 μL Chloroform:isoamyl alcohol (24:1), transfer aq. to a new tube.
- Add 2 volumes abs. ethanol, incubate on ice for 30 min., centrifuge for 10 min., wash pellet once with 200 μL 70% ethanol, spin for 5 minutes.
- Dry pellet and resuspend in ca. 40 μL H_2_O. For Southern analysis use about 1-3 μL. Add RNAase at the end of the RD.

TEN = 10 mM Tris-HCl, 10 mM EDTA, 150 mM NaCl.

SDS-EB = 2% SDS, 400 mM NaCl, 40 mM EDTA, 100 mM Tris-HCl, pH 8.0.
